# Predictors of Intestinal Parasites among Food Handlers in Goba Town, Southeast Ethiopia, 2020

**DOI:** 10.1155/2022/3329237

**Published:** 2022-06-07

**Authors:** Abate Lette, Getahun Negash, Musa Kumbi, Abduljewad Hussen, Jeylan Kassim, Demisu Zenbaba, Habtamu Gezahgn, Mitiku Bonsa, Rameto Aman, Adem Abdulkadir

**Affiliations:** ^1^Department of Public Health, Goba Referral Hospital, Madda Walabu University, Bale-Goba, Ethiopia P.O. Box 174; ^2^Department of Medical Laboratory Science, Goba Referral Hospital, Madda Walabu University, Bale-Goba, Ethiopia P.O. Box 174; ^3^Department of Public Health, Shashemene Campus, Madda Walabu University, Shashemene, Ethiopia P.O.Box; ^4^Department of Biomedical Science, Goba Referral Hospital, Madda Walabu University, Bale-Goba, Ethiopia P.O. Box 174

## Abstract

**Background:**

Globally, around 1.9 million people were dying due to food-borne diseases annually, and intestinal parasites infected one-third of the population, according to estimates and more prevalent in developing countries due to poverty. This study assessed predictors of intestinal parasites among food handlers working in Goba towns.

**Methods:**

A laboratory-based cross-sectional study was conducted from October to December 2020. Clean, dry, and leak-proof stool cups were used to collect the samples. The SPSS version 20 computer software was used to enter and clean the data, code it, and analyze it. The researchers performed binary and multivariable logistic regression analyses, with a *p* value of 0.05 considered significant.

**Result:**

A total of 98 (34%) of the 288 food workers tested positive for various intestinal parasites. *Giardia lamblia* was the most common parasite, with 42 (14.6%), followed by *Entamoeba histolytica/dispar* with 31 (10.8%), *Ascaris lumbricoides* with 8 (2.8%), *Taenia species* with 5 (1.7%), and *E. vermicularis* with 4 (1.4%). Six (2%) of the 98 positive food handlers had two infections. *E. histolytica* and *G. lamblia* were the most common parasites found in mixed infections. Hand washing with soap and water before handling food (AOR: 3.06, 95% CI: 1.16, 7.26) and untrimmed fingernail status (AOR: 2.3, 95% CI: 1.14, 4.34) were found to be strongly linked to intestinal parasite infection.

**Conclusion:**

In this investigation, intestinal parasite species were found in 34% of stool samples. Independent predictors of intestinal parasite infection were fingernail status and hand washing with water and soap use before food handling. To control intestinal parasite infection among food handlers in the research area, personal hygiene and ambient cleanliness should be improved.

## 1. Introduction

Globally, around 1.9 million people died due to food-borne diseases annually [1, 2]; and intestinal parasites infect one-third of the population, according to estimates [3]. The problems were more prevalent in developing countries due to different reasons such as poverty and lack of public health awareness [4].

In many underdeveloped nations, like Ethiopia, being infected with intestinal parasites is prevalent, owing to inadequate environmental sanitation, poor personal hygiene, and a lack of information [5–7]. With varied reports on the major parasite species and hygiene measures, the prevalence of infection with intestinal parasites among food handlers ranged from 29% to 63% [8–11].

A third of Ethiopians are infected with *A. lumbricoides*, a quarter with *T. trichiura*, and one in eight with hookworm, according to estimates. As a result, Ethiopia has the second greatest burden of *ascariasis* in sub-Saharan Africa, the third highest burden of hookworm, and the fourth highest burden of *trichuriasis* [8]. To address the public health problems caused by NTDs, Ethiopia's Federal Ministry of Health (FMoH) has emphasized intestinal parasite infection as one of the neglected tropical diseases (NTDs) in the national master plan of NTDs [3]. Therefore, this research was done to determine the predictors of intestinal parasites among food handlers working in Goba town.

## 2. Methods and Materials

### 2.1. Study Design, Area, and Period

A cross-sectional laboratory study of asymptomatic food handlers working in catering establishments in Goba town, Southeast Ethiopia, was conducted from September to December 2020. The Goba town is located 445 km from Addis Ababa, the capital city of Ethiopia, respectively. A number of food establishments and food handlers were obtained from the Culture and Tourism Office of Goba town.

All food handlers working in Goba town catering establishments were the study source population. And all food handlers from randomly selected food catering establishments in Goba town was study subjects.

All food handlers who reported or have never used medication in the last 2 weeks and during the study period and working in food catering establishments such as hotels, cafeterias, restaurants, tea/snack houses, and butchers were included in the study, and food handlers who reported to have used any drug for intestinal parasite treatment in the last 2 weeks and during the study period were excluded from the study.

### 2.2. Sample Size Determination

Using a single population proportion formula, the sample size was obtained by taking an estimated 52.1 percent proportion [5, 6], 5% margin of error (*d* = 0.05), and 95% confidence interval (*Z* ∝ /2 = 1.96) and by taking 5% nonresponse rate. *n* = (z∝/2)2∗*P*(1 − *P*)*d*2 = 383 + 19(5%) = 402.

An adjustment formula was employed when the total population of the source was 1,083 (less than 10,000) [12], and final sample size after correction was 293.

### 2.3. Data Collection and Laboratory Procedure

The study participants' sociodemographic variables were collected using a pretested and standardized questionnaire. The questionnaire was developed from different literatures [3, 4, 13]. The tool was written in English and then translated into Afan Oromo before being checked for consistency by an independent language expert. Face-to-face interviews and structured questionnaires were used to obtain data on food handlers' sociodemographic variables and personal hygiene practices; and an observational checklist was used to collect data related to the sanitary conditions of catering establishments.

For data collection and microbiological analysis, three medical laboratory personnel and three sanitarians were hired. The lead investigator trained the data collectors for two days on data and specimen collection techniques. A sampling frame was established for all food handlers prior to data collection. The final sample size was appropriately allotted to each stratum to identify representative participants. The fundamental goal of stratification was to prevent overrepresentation or underrepresentation of specific sorts of food/drinking establishments. A systematic sampling strategy was used to choose participants 4^th^ interval. The first person to answer was chosen at random. Then, from each establishment, only one food handler was chosen at random. The personal hygiene of food handlers and the hygienic conditions of food establishments were assessed using an observational checklist.

Participants were requested to provide stool samples after the interview. Clean, dry, and leak-proof stool containers were used to collect the sample.

### 2.4. Sample Processing and Identification of Intestinal Parasites

The techniques of direct wet mount and formol ether concentration were used to identify intestinal parasites. Fresh stool samples (2 mg of stool) were put on a slide with a wooden applicator, emulsified with a drop of physiological saline (0.85%) for diarrheic and semisolid samples. To detect and identify cysts of protozoan parasites in the produced stools, iodine wet mount was used. The eggs and larvae of helminthes parasites, as well as cysts and trophozoites of protozoan parasites, were then covered with a cover slide and inspected under a microscope with first ×10 objective and ×40 objectives, respectively [14]. To avoid missing *E. histolytica* and *G. lamblia* trophozoites, a direct saline wet mount was used [8].

### 2.5. Data Quality Control

SOP was closely followed during the processing of each sample to manage the quality of the work. Data consistency and completeness were ensured throughout the data collecting, input, and analysis processes.

### 2.6. Data Processing and Analysis

Using SPSS version 20 computer software, all data elements were entered, cleaned, coded, and analyzed. Descriptive statistics were used to calculate the frequency distribution, percentages, mean, and standard deviation. The analysis included binary logistic regression and multivariable logistic regression with a 95% percent confidence interval (CI). To control cofounders, binary logistic regression variables with a *p* value of 0.25 were chosen for a multivariable logistic regression model. Then, in multivariable logistic regression analysis, all variables with a *p* value of 0.05 were considered significant.

## 3. Result

### 3.1. Sociodemographic Data

The study enlisted the participation of 288 asymptomatic food handlers, with a response rate of 98.3 percent. One hundred sixty (55.6%) of the participants were females. With a standard deviation of 10.54 years, the mean age was 33.4 years. 194 (67.4%) of the 288 food handlers were between the ages of 21 and 35. The majority of those who took part in the survey had finished secondary school 114 (39.6%). More than one-third of the food handlers had from 1 to 5 years of experience. Sixty-eight percent of the participants lacked certification in food preparation and handling. Three-fourths of the respondents had no medical checkup (Table 1).

### 3.2. Prevalence of Intestinal Parasites

A total of 98 (34%) of the 288 stool samples tested positive for various intestinal parasites. *Giardia lamblia* was the most common parasite, accounting for 42 (14.6%), followed by *Entamoeba histolytica/dispar* with 31 (10.8%), *Ascaris lumbricoides* with 8 (2.8%), *Taenia species* with 5 (1.7%), and *Enterobius vermicularis* with 4 (1.4%). Six (2%) of the 98 positive food handlers had two parasitic illnesses. *Entamoeba histolytica/dispar* and *Giardia lamblia* were the most common parasites found in mixed infections (Table 2).

### 3.3. Risk Factors of Intestinal Parasites

Factors like age, sex, educational status, service year, food preparation and handling certification, checkup (medical), fingernail status, hand washing after toilet with water and soap, hand washing before food handling with water and soap, working when suffering from diseases like cough and skin, and touching food with bare hands were investigated to see if they were linked to parasite infection in the study subjects.

Hand washing with water and soap before handling food and fingernail status were found to be strongly linked to intestinal parasite infection. Parasite infections were 2.3 times more likely to occur (AOR: 2.3, 95% CI: 1.14, 4.34) among food workers who had untrimmed fingernails compared to those who trimmed them, according to the multivariable logistic analysis results. Food handlers who did not wash their hands with water and soap before handling food were 3.06 times (AOR: 3.06, 95% CI: 1.16, 7.26) more likely to become infected with an intestinal parasite than those who did (Table 3).

## 4. Discussion

In this investigation, intestinal parasites were found in 34 percent of the food handlers. The high prevalence of intestinal parasites among food handlers in this study was consistent with earlier Ethiopian investigations, such as in food handlers at Arba Minch University (36%) [14]. In Yebu town, Southwest Ethiopia, a higher frequency of intestinal parasites was reported (44.1%) [8], Nekemte town (52.1%) [5], and Gonder, Northwest Ethiopia (60.2%) [15] and southern Ethiopia [12]. However, compared to the current study, Northwest Ethiopia had a lower prevalence (29.1%) [1] and Sari, Northern Iran (15.5%) [2]. This disparity could be attributable to a variety of factors, including epidemiological differences, environmental distribution differences, inadequate personal hygiene practices, environmental cleanliness, and a lack of knowledge about health promotion behaviors.

The parasites *Entamoeba histolytica/dispar* and *Giardia lamblia* were found to be the most frequently in mixed infections, according to the study (1%). This research backs up a study conducted in a community school near the Haramaya University, Eastern Ethiopia, which found that 1% of students had combined infections of *Entamoeba histolytica/dispar* and *Giardia lamblia* [13]. This is due to a resemblance in the cleanliness of the environment.

In this study, fingernail status, hand washing with water and soap before food handling, and hand washing with water and soap after toilet had a statistically significant association with parasite infection. When compared to their counterparts, food handlers with untrimmed fingernails were 2.3 times more likely to contract parasites (AOR: 2.3, 95 % CI: 1.14, 4.34). This finding was supported by research from Central Ethiopia [16], in which trimming of fingernails was a factor associated with intestinal parasite infestation [5], and Yebu town, Southwest Ethiopia [8]. This is helpful in designing advocacy methods for personal hygiene in the study area and country as well.

Food handlers who did not wash their hands with water and soap before handling food were 3.06 times (AOR: 3.06, 95% CI: 1.16, 7.26) more likely to be infected with intestinal parasites than those who did. Similarly, in Southwest Ethiopia, food handlers who did not follow regular hand washing before a meal were an independent predictor of intestinal parasite infection [8], and in Southern Ethiopia, hand washing before food handling was associated with parasite infection [14]. When compared to other studies, this clearly suggests that hand hygiene habits and advocacy among food handlers should be implemented in the study location.

## 5. Conclusions and Recommendations

The prevalence of intestinal parasites among food handlers was found to be high in this study. Intestinal parasite infection was predicted by fingernail status and hand washing with water and soap before food handling, both of which were independent predictors. To control intestinal parasites among food handlers in the research area, personal cleanliness and environmental sanitation were recommended. In addition, advocating for hand hygiene and personal hygiene for food handlers in various settings is encouraged.

## Figures and Tables

**Table 1:** Sociodemographic characteristics of food handlers (*N* = 288) working in Goba town at food service establishments, 2020.

Variables	Frequency	Percentage
Age (in years)		
≤20	63	21.9
21-35	194	67.4
>35	31	10.6
Sex		
Male	128	43.7
Female	160	55.6
Service year		
<1	11	3.8
1-5	103	35.2
6-10	92	31.9
>10	82	28.5
Educational status		
Illiterate	21	7.2
Primary school	114	39.6
Secondary school	100	34.7
Higher than secondary school	53	18.4
Certification in food preparation and handling		
Yes	92	31.9
No	196	68.1
Checkup (medical check at health facility)		
Yes	70	24.3
No	218	75.7

**Table 2:** Intestinal parasite prevalence among food handlers (*N* = 288) working in Goba town, 2020.

Type of parasites	Frequency	Percentage
Single infection		
*Giardia lamblia*	42	14.6%
Cyst of *Giardia lamblia*	12	4.2%
Trophozoite of *Giardia lamblia*	30	10.4%
*Entamoeba histolytica/dispar*	31	10.8%
Trophozoite of *Entamoeba histolytica/dispar*	24	8.3%
Cyst of *Entamoeba histolytica/dispar*	7	2.4%
*Schistosoma mansoni*	2	0.7%
*Enterobius vermicularis*	4	1.4%
*Ascaris lumbricoides*	8	2.8%
*Taenia species*	5	1.7%
Double infection		
Cyst of *Giardia lamblia* and cyst of *Entamoeba histolytica/dispar*	3	1%
*Taenia saginata* and cyst of *Giardia lamblia*	1	0.3%
Cyst of *Giardia lamblia* and Trophozoite of *Entamoeba histolytica/dispar*	2	0.7%

Cyst of *Giardia lamblia* and cyst of *Entamoeba histolytica/dispar 3(1%).*

**Table 3:** Multivariate logistic regression analysis of predictors (personal hygiene variables) for parasite infection among food handler's working in Goba town, Ethiopia, 2020 (*n* = 288).

Variables	Infection		COR (95% CI)	AOR (95% CI)
	Negative	Positive		
Fingernail status				
Trimmed	90 (31.2)	29 (10.1)	1	1
Not trimmed	100 (34.7)	69 (23.9)	5.9 (2.52, 8.55)^∗^	2.3 (1.14, 4.34)^∗^
Hand washing after toilet with water and soap				
Yes	107 (37.1)	11 (3.8)	1	1
No	83 (28.8)	87 (30.2)	4.4 (2.14, 19.30)^∗^	4.6 (0.16, 12.09)
Hand washing before food handling with water and soap				
Yes	98 (34)	23 (8)	1	1
No	92 (31.9)	76 (26.3)	3.19 (2.52, 8.55)^∗^	3.06 (1.16, 7.26)^∗^
Working when suffering from diseases like cough and skin				
Yes	112 (38.9)	34 (11.8)	1	1
No	78 (27.1)	64 (22.2)	0.6 (0.16, 0.89)^∗^	0.5 (0.34, 9.05)

*Note*: statistically significant at *p* value < 0.05, 1 = reference.

## Data Availability

On request, data and resources will be made available. If you require data and resources at any time, please contact Mr. Adem Abdulkadir.
